# Adult vaccination coverage levels among users of complementary/alternative medicine – results from the 2002 National Health Interview Survey (NHIS)

**DOI:** 10.1186/1472-6882-8-6

**Published:** 2008-02-22

**Authors:** Shannon Stokley, Karen A Cullen, Allison Kennedy, Barbara H Bardenheier

**Affiliations:** 1 Immunization Services Division, National Center for Immunization and Respiratory Diseases, Centers for Disease Control and Prevention, Atlanta, GA, USA; 2 Division of Health Care Statistics, National Center for Health Statistics, Centers for Disease Control and Prevention, Hyattsville, MD, USA

## Abstract

**Background:**

While many Complementary/Alternative Medicine (CAM) practitioners do not object to immunization, some discourage or even actively oppose vaccination among their patients. However, previous studies in this area have focused on childhood immunizations, and it is unknown whether and to what extent CAM practitioners may influence the vaccination behavior of their adult patients. The purpose of this study was to describe vaccination coverage levels of adults aged ≥ 18 years according to their CAM use status and determine if there is an association between CAM use and adult vaccination coverage.

**Methods:**

Data from the 2002 National Health Interview Survey, limited to 30,617 adults that provided at least one valid answer to the CAM supplement, were analyzed. Receipt of influenza vaccine during the past 12 months, pneumococcal vaccine (ever), and ≥ 1 dose of hepatitis B vaccine was self-reported. Coverage levels for each vaccine by CAM use status were determined for adults who were considered high priority for vaccination because of the presence of a high risk condition and for non-priority adults. Multivariable analyses were conducted to evaluate the association between CAM users and vaccination status, adjusting for demographic and healthcare utilization characteristics.

**Results:**

Overall, 36% were recent CAM users. Among priority adults, adjusted vaccination coverage levels were significantly different between recent and non-CAM users for influenza (44% vs 38%; p-value < 0.001) and pneumococcal (40% vs 33%; p-value < 0.001) vaccines but were not significantly different for hepatitis B (60% vs 56%; p-value = 0.36). Among non-priority adults, recent CAM users had significantly higher unadjusted and adjusted vaccination coverage levels compared to non-CAM users for all three vaccines (p-values < 0.001).

**Conclusion:**

Vaccination coverage levels among recent CAM users were found to be higher than non-CAM users. Because CAM use has been increasing over time in the U.S., it is important to continue monitoring CAM use and its possible influence on receipt of immunizations among adults. Since adult vaccination coverage levels remain below Healthy People 2010 goals, it may be beneficial to work with CAM practitioners to promote adult vaccines as preventive services in keeping with their commitment to maintaining good health.

## Background

Complementary and alternative medicine (CAM) is the collective term for treatments and therapies not routinely offered by mainstream medical practitioners, or widely taught at U.S. medical schools [1]. This broad definition encompasses a range of both provider and non provider-based interventions, from chiropractic and homeopathy to herbs and nutritional supplements. A recent analysis of the Alternative Health/Complementary and Alternative Medicine supplement of the 2002 National Health Interview Survey (NHIS) found that 75% of adults in the U.S. have ever used some type of CAM therapy, including prayer specifically for health reasons [2]. There is also evidence to suggest that the proportion of U.S. adults who report CAM use has been increasing over time [3].

Reasons for CAM use vary, but generally fall into two categories which reflect both the complementary and alternative nature of these therapies. Some people use CAM as a complement to their regular medical care, either to improve general health and wellness or as an adjunct to treatment for chronic illnesses like back pain [4]. Others prefer CAM as an alternative to mainstream medicine, citing dissatisfaction with mainstream medicine as their primary reason for use [4]. Whatever the reason for its use, many of those who use CAM in addition to conventional medical care do not report CAM use to their primary care physician [3,5]. This creates the potential for conflict, as CAM therapies may negatively interact with treatment, or contradictory advice from CAM practitioners may influence acceptance of or adherence to medical advice.

Influenza, pneumococcal, and hepatitis B vaccines are all recommended for certain segments of the adult population, depending on age and risk factors for each disease [6]. These illnesses represent a substantial burden of vaccine-preventable disease in the United States; however, influenza and pneumococcal immunization rates for target groups remain below Healthy People 2010 goals, and achievement of high hepatitis B vaccination rates among high risk adults remains a challenge [7,8]. Poor access to healthcare, low awareness of recommendations, and safety concerns are all reasons for undervaccination among adults, while physician recommendation has been cited as an important factor in acceptance of adult immunization [9-11].

It has been suggested that while many CAM practitioners do not object to immunization, some discourage or even actively oppose vaccination among their patients [12-14]. However, previous studies in this area have focused on childhood immunizations, and it is unknown whether and to what extent CAM practitioners may negatively influence the vaccination behavior of their adult patients. Conversely, adult CAM users may be more likely to accept vaccination, as CAM use may act as a signal that an individual is highly engaged in maintaining their health, and therefore more motivated to accept preventive services. Determining which is the most likely of these two scenarios is of significant public health importance given the growing popularity of CAM therapies and the continued burden of vaccine-preventable disease among U.S. adults. Therefore, the objectives of this study were to: 1) describe the influenza, pneumococcal, and hepatitis B immunization coverage levels of adults 18 years and older in the United States according to their CAM use status and 2) determine if there is an association between CAM use and immunization coverage among priority and non-priority adults for each vaccine. To our knowledge, this is the first nationally representative study to examine the association between CAM use and immunization behavior among adults in the U.S.

## Methods

### National Health Interview Survey (NHIS)

The NHIS is an annual survey conducted by the National Center for Health Statistics (NCHS), Centers for Disease Control and Prevention (CDC) that collects health information on the U.S. civilian, non-institutionalized household population. The sampling design employs a multistage area probability design; non-Hispanic black and Hispanic populations are oversampled to allow for more precise estimates of health status in these groups. In 2002, the Alternative Health/Complementary and Alternative Medicine (CAM) supplement was administered to one randomly selected adult per family. The supplemental questionnaire included questions on 27 types of CAM therapies commonly used in the United States. A complete description of the supplement can be found elsewhere [2,15]. The final sample adult response rate was 73.4%.

### Alternative Health/Complementary and Alternative Medicine

Analysis was limited to the 30,617 adults that provided at least one valid answer to the CAM supplement [16]. CAM therapies were classified according to Barnes et al [2] as practitioner-based (i.e. acupuncture; ayurveda; biofeedback; chelation therapy; chiropractic care; folk medicine; hypnosis; massage; naturopathy; and energy healing therapy/Reiki) and non-practitioner-based (i.e. natural herbs such as ginger, Echinacea, or black cohosh; homeopathic treatment; high dose or megavitamin therapy; special diets such as vegetarian, macrobiotic, Atkins, Pritikin, Ornish, and Zone; Yoga/Tai Chi/Qi Gong; and relaxation techniques such as meditation, guided imagery, progressive relaxation, and deep breathing exercises). Respondents who indicated they had used any of the previously listed CAM therapies during the past 12 months were considered 'Recent' CAM users; respondents who indicated they have used one or more CAM therapies but not in the past 12 months were considered 'Past' CAM users; 'Never' CAM users included persons who indicated they have never used any CAM therapies.

### Vaccination Status

The 2002 NHIS included questions in the adult questionnaire about three vaccines: influenza, pneumococcal, and hepatitis B. Respondents were asked if they had received a 'flu shot' during the past 12 months, if they had ever received a 'pneumonia shot', and if they had ever received a dose of the hepatitis B vaccine. Vaccination status was self reported by the respondent and was not verified by a provider. Respondents who answered "Don't know" or refused to answer the vaccination questions (0.5% for influenza vaccine; 2.7% for pneumococcal vaccine; 5.6% for hepatitis B vaccine) were excluded from the analysis for the vaccine of interest.

For each vaccine, respondents were categorized as 'priority' or 'non-priority' adults according to vaccine recommendations from the Advisory Committee on Immunization Practices [17-19]. A list of the criteria used to categorize adults as 'priority' can be seen in Table 1. All responses to NHIS questions regarding health care conditions and occupation are self reported by the respondent.

**Table 1:** Criteria used for classifying adults as high priority for receiving select vaccines

**Vaccine**	**Criteria**
Influenza	Age ≥ 50 years; ever had diabetes; ever had coronary heart disease, angina, myocardial infarction or some other heart condition; ever had emphysema, chronic bronchitis, or an asthma attack within the past 12 months; or, was diagnosed with cancer within the past 12 months
Pneumococcal	Age ≥ 65 years; ever had diabetes; ever had coronary heart disease, angina, myocardial infarction or some other heart condition; ever had emphysema or chronic bronchitis; had weak kidneys during the past 12 months; had a liver condition in the past 12 months; or, was diagnosed with cancer within the past 12 months
Hepatitis B	Was in a healthcare, police or fire occupation; reported having had a sexually transmitted infection other than HIV in the last 5 years; considered their risk of getting HIV as "high/have AIDS" or "medium high"; or, they answered yes to being in any of the six hepatitis B risk groups (having hemophilia and received clotting factor concentrates, men who have had sex with men, ever used street drugs by needle, ever traded sex for money or drugs, ever tested positive for HIV, or ever had sex with someone in any of these groups)

### Data analysis

Statistical analysis included estimating the proportion and 95% confidence intervals of adults who are recent, past, or never CAM users by demographic characteristics. Estimated vaccination coverage levels for priority and non-priority adults for influenza, pneumococcal, and hepatitis B vaccines were also assessed by CAM use status (unadjusted coverage levels). To evaluate the association between CAM use and vaccination status, multivariable analyses were conducted which adjusted for the demographic and healthcare utilization characteristics listed in Table 2. Separate models were created for priority and non-priority adults for each of the three vaccines of interest (adjusted coverage levels). Independent variables were tested for joint collinearity using condition indices, a measure of multi-collinearity. Evaluation of condition indices indicated little collinearity among variables in the logistic regression models. Because previous studies have suggested that some Chiropractors oppose vaccination [14], we also evaluated vaccination coverage levels among Chiropractic users.

**Table 2:** Demographic characteristics and vaccination status by Complementary/Alternative Medicine (CAM) use, 2002 National Health Interview Survey

**Characteristic**	**Recent CAM user (N = 10,867)**		**Past CAM user (N = 12,781)**		**Never CAM user (N = 6969)**	
	**#**	**% (95% CI)**	**#**	**% (95% CI)**	**#**	**% (95% CI)**
**Sex**						
Female	6727	58.2 (± 1.1)	7154	51.3 (± 1.1)	3430	43.5 (± 1.4)
**Age (years)**						
18–49 yrs	6834	65.0 (± 1.1)	7027	58.1 (± 1.2)	4550	68.1 (± 1.4)
50–64 yrs	2545	23.4 (± 0.9)	2725	21.8 (± 0.9)	1164	16.3 (± 1.0)
65+ yrs	1488	11.6 (± 0.7)	3029	20.0 (± 0.9)	1255	15.6 (± 0.9)
**Race/ethnicity**						
NH-White	7686	76.8 (± 1.0)	8827	75.8 (± 1.1)	3532	60.5 (± 1.7)
NH-Black	1117	8.5 (± 0.7)	1632	10.6 (± 0.7)	1285	16.5 (± 1.3)
Hispanic	1459	8.5 (± 0.6)	1844	9.3 (± 0.7)	1904	18.7 (± 1.2)
NH-Other	605	6.2 (± 0.6)	478	4.4 (± 0.5)	248	4.4 (± 0.7)
**Region**						
Northeast	2009	19.6 (± 1.0)	2333	19.1 (± 1.0)	1241	18.7 (± 1.3)
Midwest	2675	25.8 (± 1.4)	3091	25.6 (± 1.1)	1291	20.5 (± 1.6)
South	3460	31.6 (± 1.4)	4867	38.1 (± 1.2)	3000	43.6 (± 1.8)
West	2723	23.0 (± 1.4)	2490	17.2 (± 0.9)	1437	17.2 (± 1.2)
**Education level**						
Less than High School	1186	9.5 (± 0.7)	2342	15.9 (± 0.8)	2335	30.1 (± 1.4)
High School or GED^a^	2570	24.8 (± 1.1)	4021	32.4 (± 1.0)	2152	33.3 (± 1.3)
More than High School	7061	65.7 (± 1.2)	6310	51.7 (± 1.1)	2301	36.6 (± 1.6)
**Poverty Status**						
Below poverty	985	6.5 (± 0.5)	1328	7.6 (± 0.6)	1088	12.1 (± 0.9)
At or above poverty, < 200% poverty	1375	10.7 (± 0.7)	1915	12.7 (± 0.7)	1284	16.6 (± 1.0)
At or above 200% poverty	6486	63.9 (± 1.1)	6419	55.0 (± 1.1)	2502	40.8 (± 1.5)
Unknown	2021	18.8 (± 0.9)	3119	24.7 (± 1.0)	2095	30.5 (± 1.5)
**Smoking status**						
Current	2435	22.0 (± 1.0)	2727	21.3 (± 0.9)	1708	26.0 (± 1.3)
Former	2705	25.4 (± 1.1)	2953	23.5 (± 0.8)	1081	16.3 (± 1.1
Never	5706	52.6 (± 1.2)	7046	55.2 (± 1.0)	4047	57.6 (± 1.6)
**# Dr. visits**						
0–1 visits	2933	27.2 (± 1.0)	4400	35.3 (± 1.0)	3394	50.2 (± 1.5)
2–9 visits	5659	53.2 (± 1.0)	6560	52.3 (± 1.1)	2782	40.9 (± 1.5)
10+ visits	2188	19.6 (± 0.9)	1698	12.4 (± 0.6)	666	8.9 (± 0.8)
**Usual source of care**^b^						
Yes	9454	87.6 (± 0.7)	11121	88.0 (± 0.7)	5280	77.5 (± 1.2)
**Priority for influenza vaccine**						
Yes	5211	45.9 (± 1.2)	6627	49.0 (± 1.2)	2852	38.5 (± 1.5)
**Priority for pneumococcal vaccine**						
Yes	3238	27.5 (± 1.0)	4631	33.0 (± 1.1)	1968	26.0 (± 1.3)
**Priority for hepatitis B vaccine**						
Yes	1407	12.5 (± 0.7)	1052	8.3 (± 0.6)	498	6.8 (± 0.7)
**Insurance status**						
No Insurance	1537	13.2 (± 0.8)	1755	12.7 (± 0.7)	1782	24.2 (± 1.2)
Medicaid Only	447	3.1 (± 0.4)	595	3.8 (± 0.4)	600	7.0 (± 0.7)
Medicare Only	387	3.0 (± 0.4)	854	5.4 (± 0.5)	466	5.5 (± 0.6)
Single, Military, Other Pub/Govt, or IHS Only	229	1.8 (± 0.3)	288	2.0 (± 0.3)	160	2.1 (± 0.4)
Private Only	6217	61.0 (± 1.2)	6390	55.2 (± 1.1)	2932	47.1 (± 1.6)
Multiple Insurance	2050	17.8 (± 0.8)	2899	20.8 (± 0.9)	1029	14.0 (± 0.9)
**Received influenza vaccine**	3189	28.7 (± 1.0)	4165	31.4 (± 0.9)	1441	20.0 (± 1.1)
**Received pneumococcal vaccine**	1788	16.1 (± 0.8)	2513	18.4 (± 0.8)	771	10.9 (± 0.8)
**Received hepatitis B vaccine**	3066	30.1 (± 1.1)	2799	24.0 (± 1.0)	1206	19.5 (± 1.3)

^a^GED: General Educational Development

^b^From the question: "Is there a place that you usually go to when you are sick or need advice about your health?"

Because NHIS is a complex survey that involves clustering, stratification, and multistage sampling techniques to obtain nationally representative information on the health of the nation, all analyses were conducted using SAS, release 8.0 (SAS Institute, Cary, NC, 2001) and SUDAAN, release 9 (Research Triangle Institute, Research Triangle Park, NC, 2001) to account for the survey sample design in calculating the standard errors and confidence intervals. The data file obtained from the NCHS included the sample weights used in the analysis [16]. Categorical independent variables were compared using the Chi-square test. Multiple logistic regression and computation of predictive margins were used to estimate the probability of vaccination when controlling for all other independent variables. The predictive margin for each variable included in the model along with the corresponding 95% confidence interval was reported. Predictive margins are a type of direct standardization that allows for the comparison of group outcomes while controlling for the covariate distribution in the population. They are best used when the outcome is not rare (i.e. > 10%). They are easier to interpret than odds ratios and allow for different comparisons to be made, as no referent group is designated [20,21].

## Results

### CAM Use

Overall, 35.7% (± 0.7%) of adults 18 years and older were recent CAM users, 42.6% (± 0.7%) were past CAM users, and 21.7% (± 0.7%) were never CAM users. Among recent CAM users, 13.6% (± 0.8%) used practitioner based therapies, 66.9% (± 1.1%) used non-practitioner based therapies, and 19.5% (± 0.9%) used both practitioner and non-practitioner based therapies; 69.4% (± 1.1%) reported that CAM use was somewhat or very important to maintaining their health. Demographic characteristics of recent, past, and never CAM users can be seen in Table 2. Recent CAM users had a higher frequency of being female, of being non-Hispanic white, living in the West Census region, having greater than a high school education level, living at or above 200% of the federal poverty level, making 10 or more visits to the doctor during the past 12 months, being in the priority group for hepatitis B vaccine, and having only private health insurance.

### Immunization Status

#### Unadjusted vaccination coverage

Among priority adults, overall vaccination coverage levels were 43.1% (± 1.0%) for influenza, 39.0% (± 1.2%) for pneumococcal, and 57.5% (± 2.2%) for hepatitis B vaccines. Unadjusted vaccination coverage levels by CAM use can be seen in Table 3. Never CAM users had significantly lower vaccination coverage levels for influenza, pneumococcal, and hepatitis B vaccines compared to recent and past CAM users. Past CAM users had significantly higher vaccination coverage levels for influenza and pneumococcal vaccines when compared to recent CAM users.

**Table 3:** Unadjusted and adjusted^a ^vaccination coverage levels among priority and non-priority adults by CAM use, 2002 National Health Interview Survey

	**Influenza % (95% CI)**	**Pneumococcal % (95% CI)**	**Hepatitis B % (95% CI)**
**Priority Adults**	(n = 14,595)	(n = 9,596)	(n = 2,848)
Unadjusted results			
CAM use			
Recent	42.1 (40.6, 43.6)	37.6 (35.6, 39.5)	59.9 (56.7, 63.1)
Past	46.9 (45.5, 48.3)*	43.6 (42.0, 45.2)*	58.2 (54.8, 61.7)
Never	35.6 (33.4, 37.7)*^+^	30.1 (27.8, 32.4)*^+^	48.4 (42.8, 54.0)*^+^
			
Adjusted results			
CAM use			
Recent	43.5 (41.9, 45.1)	39.6 (37.6, 41.6)	59.5 (56.4, 62.6)
Past	44.4 (43.0, 45.8)	41.2 (39.6, 42.8)	58.2 (54.9, 61.5)
Never	38.2 (36.0, 40.4)*^+^	32.7 (30.3, 35.1)*^+^	56.4 (50.9, 61.9)
			
**Non-Priority Adults**	(n = 15,927)	(n = 20,328)	(n = 26,179)
Unadjusted results			
CAM use			
Recent	17.3 (16.1, 18.5)	8.0 (7.2, 8.7)	25.7 (24.6, 26.9)
Past	16.4 (15.4, 17.5)	6.0 (5.4, 6.6)*	20.9 (19.8, 21.9)*
Never	10.3 (9.2, 11.4)*^+^	4.1 (3.5, 4.8)*^+^	17.4 (16.0, 18.7)*^+^
			
Adjusted results			
CAM use			
Recent	16.1 (14.9, 17.3)	7.6 (6.8, 8.4)	27.5 (26.1, 28.9)
Past	16.3 (15.3, 17.3)	6.0 (5.4, 6.6)*	25.6 (24.4, 26.8)*
Never	12.0 (10.6, 13.4)*^+^	4.4 (3.6, 5.2)*^+^	21.1 (19.5, 22.7)*^+^

* denotes significantly different than recent cam users; + denotes significantly different than past cam users

^a^Multivariable model adjusts for age, gender, race/ethnicity, education level, federal poverty level, region, having a usual source of care other than the emergency department, number of visits to a physician during the past 12 months, health insurance status, and smoking status.

Among non-priority adults, overall vaccination coverage levels were 15.3% (± 0.7%) for influenza, 6.3% (± 0.4%) for pneumococcal, and 21.8% (± 0.8%) for hepatitis B vaccines. Unadjusted vaccination coverage levels by CAM use can be seen in Table 3. Recent CAM users had significantly higher pneumococcal and hepatitis B vaccination coverage levels compared to past CAM users and significantly higher coverage for all three vaccines compared to never CAM users. The largest difference in coverage was observed for the hepatitis B vaccine, where recent CAM users had coverage levels 8.3 percentage points higher than never CAM users.

#### Adjusted vaccination coverage

In multivariable analyses adjusting for demographic and health care utilization characteristics, the difference in hepatitis B coverage levels for priority adults between recent and never CAM users was no longer statistically significant (Table 3). For non-priority adults, adjusted differences between recent, past and never CAM users remained significant for influenza, pneumococcal, and hepatitis B vaccines.

#### Vaccination coverage among Chiropractic users

Overall, 19.8% (± 0.6%) of respondents reported ever using Chiropractic services; 7.5% (± 0.4%) reported visiting a chiropractor in the past 12 months and 12.3% (± 0.5%) reported visiting a chiropractor more than 12 months ago. Because of the small sample sizes among the recent and past chiropractic use categories, we combined the groups for the vaccination coverage analysis which can be seen in Table 4. In general, persons who have reported ever using chiropractic services had lower vaccination coverage levels than non-chiropractic CAM users. However, among priority adults, chiropractic users had significantly higher vaccination coverage levels for influenza and pneumococcal vaccines compared to never CAM users. Among non-priority adults, chiropractic users had significantly higher vaccination coverage levels for all vaccines compared to never CAM users.

**Table 4:** Adjusted^a ^vaccination coverage levels of priority and non-priority adults by Chiropractic therapy use, 2002 National Health Interview Survey

	**Influenza % (95% CI)**	**Pneumococcal % (95% CI)**	**Hepatitis B % (95% CI)**
**Priority Adults**	(n = 14,595)	(n = 9,596)	(n = 2,848)
Chiropractic use			
Use Chiropractic therapy	41.7 (39.9, 43.5)	40.3 (39.3, 42.7)	55.2 (50.3, 60.1)
CAM user, non-chiropractic	44.9 (43.7, 46.1)*	40.7 (39.3, 42.1)	60.1 (57.6, 62.6)
Never used CAM	38.3 (36.1, 40.5)*^+^	32.6 (30.2, 35.0)*^+^	56.7 (51.2, 62.2)
			
**Non-Priority Adults**	(n = 15,927)	(n = 20,328)	(n = 26,179)
Chiropractic use			
Use Chiropractic therapy	15.8 (14.2, 17.4)	6.9 (6.1, 7.7)	25.7 (23.9, 27.4)
CAM user, non-chiropractic	16.3 (15.3, 17.3)	6.7 (6.1, 6.7)	26.8 (25.8, 27.8)
Never used CAM	12.0 (10.6, 13.4)*^+^	4.5 (3.7, 5.3)*^+^	21.2 (19.6, 22.8)*^+^

* denotes significantly different than chiropractic users; + denotes significantly different than non-chiropractic CAM users

^a^Multivariable model adjusts for age, gender, race/ethnicity, education level, federal poverty level, region, having a usual source of care other than the emergency department, number of visits to a physician during the past 12 months, health insurance status, and smoking status.

## Discussion

Results from a nationally representative survey of U.S. adults have shown that after adjusting for demographic and healthcare utilization characteristics, coverage levels for influenza and pneumococcal vaccines were significantly higher among priority adults who were recent or past CAM users compared to adults who had never used a CAM therapy. Among non-priority adults, adjusted coverage levels for influenza, pneumococcal, and hepatitis B vaccines were significantly higher among recent or past CAM users when compared to never CAM users. Additionally, adults using chiropractic services had higher vaccination coverage levels compared to never CAM users.

Results from this study are somewhat unexpected considering findings from previous studies have shown that parents with an alternative medical orientation were significantly less accepting of immunizations for their children [22,23]. A national survey of parents of children aged ≤ 6 years found that when compared to parents with a conventional medical orientation, parents with an alternative medical orientation were significantly less likely to believe immunizations are important (89.4% vs. 75.5%, respectively) and more likely to choose to opt out of an immunization for their child (11.2% vs. 24.9%, respectively) [22]. In a study of parents of school children, parents claiming vaccine exemptions for their children were more likely than parents of vaccinated children to report use of CAM professionals by their immediate family members (79.6% vs. 51.2%, respectively) [23]. It could be that parents are more cautious of vaccination for their children because the hypothesized risks for children's immunizations are more publicized and more severe [24] than those for adult immunizations. Also, parents may be more averse to risks that are involuntary (e.g., school entry laws), and risks to children (that cause greater fear or dread than risks to adults) [25,26].

Our results suggest that, similar to previous studies, respondents use CAM as supplemental rather than alternative health care [4,27]. Recent and past CAM users in our study were significantly more likely to have more doctor visits than non-CAM users. Because CAM users tend to have more doctor visits, they likely have more contact with physicians which provide more opportunities to be immunized. The positive association between CAM use and immunization coverage among priority adults also suggests that even if CAM users are receiving negative information regarding immunizations from their CAM providers, it is not affecting their receipt of immunizations at this time.

The difference in hepatitis B vaccination coverage levels between recent and non-CAM users was relatively small (i.e. 4–6 percentage points for priority and non-priority adults) and remained significant among non-priority adults after adjusting for demographic and healthcare utilization characteristics. The finding that CAM users had higher vaccination coverage levels for hepatitis B has several possible explanations. First, CAM users might be more concerned about their general health and wellness [4] and therefore more likely to get all immunizations. Another possible reason could be that CAM providers request their patients to receive the hepatitis B vaccination to protect themselves from liability in light of publicized hepatitis B outbreaks among alternative medicine practices in which CAM procedures required injections [28,29]. Notably, many CAM therapies are available for persons with hepatitis B or hepatitis C disease, and HIV. Thus populations at high risk for hepatitis B infection might already be aware of the healing opportunities CAM therapies may provide. In addition, hepatitis B is a sexually transmitted disease and thus those at risk for hepatitis B may also be at risk for other STDs (e.g., HIV, chronic vaginal symptoms). Studies have shown that persons with some STDs use CAM therapies to alleviate symptoms before they are diagnosed [30,31].

This study was subject to several limitations. The definition of CAM use was wide and included therapies that some may consider mainstream and not alternative, such as yoga or special diets like Atkins. The inclusion of these more common and mainstream therapies could have lessened the difference seen in vaccination coverage between CAM users and non-CAM users. Thus, the actual difference may be larger than observed. Immunization status was self-reported and not verified by a medical provider; however, a previous study found that self-report of influenza and pneumococcal vaccinations are highly sensitive, with moderate to low specificity, respectively [32]. Due to the cross sectional nature of the study we could not assess a temporal relationship and therefore we are not able to establish causal relationships. It is unknown if vaccinated respondents received their vaccines prior to or after initiating a CAM therapy.

## Conclusion

To our knowledge, this is the first nationally representative study to investigate a potential association of CAM use among adults and the receipt of vaccination. Because CAM use has been increasing over time in the U.S., it is important to continue to monitor CAM use and its possible influence on receipt of immunizations among adults. Since one-third of CAM users receive practitioner-based therapies, and adult vaccination coverage levels remain below Healthy People 2010 goals, it may be beneficial to work with CAM practitioners to promote adult vaccines as preventive services in keeping with their commitment to maintaining good health. However, before this is undertaken, a thorough understanding of the attitudes and beliefs of CAM practitioners regarding adult vaccination should be assessed.

## Competing interests

The author(s) declare that they have no competing interests.

## Authors' contributions

SS contributed to study design, data analysis and interpretation, and manuscript development. KAC contributed to study design, data interpretation, and manuscript development. AK contributed to study design, data interpretation, and manuscript development. BHB contributed to study design, data interpretation and manuscript development. All authors read and approved the final manuscript.

## Pre-publication history

The pre-publication history for this paper can be accessed here:


